# Protection from the 2009 H1N1 Pandemic Influenza by an Antibody from Combinatorial Survivor-Based Libraries

**DOI:** 10.1371/journal.ppat.1000990

**Published:** 2010-07-08

**Authors:** Arun K. Kashyap, John Steel, Adam Rubrum, Angeles Estelles, Raffaella Briante, Natalia A. Ilyushina, Li Xu, Ryann E. Swale, Aleksandr M. Faynboym, Pamela K. Foreman, Michael Horowitz, Lawrence Horowitz, Richard Webby, Peter Palese, Richard A. Lerner, Ramesh R. Bhatt

**Affiliations:** 1 Sea Lane Biotechnologies, Menlo Park, California, United States of America; 2 Departments of Microbiology and Medicine, Mount Sinai School of Medicine, New York, New York, United States of America; 3 Infectious Diseases, St. Jude Research Hospital, Memphis, Tennessee, United States of America; 4 Department of Chemistry, The Scripps Research Institute, La Jolla, California, United States of America; University of Wisconsin-Madison, United States of America

## Abstract

Influenza viruses elude immune responses and antiviral chemotherapeutics through genetic drift and reassortment. As a result, the development of new strategies that attack a highly conserved viral function to prevent and/or treat influenza infection is being pursued. Such novel broadly acting antiviral therapies would be less susceptible to virus escape and provide a long lasting solution to the evolving virus challenge. Here we report the *in vitro* and *in vivo* activity of a human monoclonal antibody (A06) against two isolates of the 2009 H1N1 pandemic influenza virus. This antibody, which was obtained from a combinatorial library derived from a survivor of highly pathogenic H5N1 infection, neutralizes H5N1, seasonal H1N1 and 2009 “Swine” H1N1 pandemic influenza *in vitro* with similar potency and is capable of preventing and treating 2009 H1N1 influenza infection in murine models of disease. These results demonstrate broad activity of the A06 antibody and its utility as an anti-influenza treatment option, even against newly evolved influenza strains to which there is limited immunity in the general population.

## Author Summary

Influenza viruses constantly challenge our ability to prevent and treat their resulting infection. From a survivor of the H5N1 influenza we have discovered an antibody that is effective against both H5N1 and seasonal H1N1 influenza viruses. Here we show the antibody is effective against 2009 pandemic influenza in a cell culture assay and also in mouse models of disease when given before and even after lethal influenza infection. The present work demonstrates the viability of this particular antibody and the general approach of using antibodies against viral pathogens as opposed to traditional treatments that are losing their efficacy for the prevention and treatment of influenza infection. We conclude the efficacy of this antibody warrants further experimental testing as an alternative therapy for treatment in man.

## Introduction

Controlling the spread of influenza remains a major challenge due to the unpredictable nature of the virus. Recently, a novel human adapted H1N1 virus has emerged and progressed globally such that the World Health Organization (WHO) has declared the first influenza pandemic in 40 years [1], [2]. Globally, efforts have been undertaken to produce vaccines and stockpile small molecule antiviral reserves to prevent and treat widespread influenza disease. While these strategies are effective, they are not without limitations. Vaccines have not provided lasting immunity against influenza because of viral mutation (“antigenic drift”) and reassortment (“antigenic shift”) [3], [4], [5], [6]. Popular small molecule antiviral treatments (oseltamivir) have recently lost effectiveness due to the rapid proliferation of seasonal H1N1 strain resistanc, demonstrating the urgent need to develop novel treatments for influenza infection and disease.

Such new treatment options would ideally be both broadly protective and provide a novel mechanism of attack against the virus. Antibodies have very desirable properties as prophylactic and therapeutic agents: long serum half-life, low immunogenicity and high specificity for antigens. In addition, antibodies are currently being used against infectious disease. For example, antibody clinical prophylaxis against RSV is a standard of care and antibody therapy is in development for treatment of anthrax [7], [8], [9], [10]. A related passive immunity strategy against influenza was used in the past during times of crisis, and retrospective studies have quantified the benefits of such strategies [11]. Furthermore, it would be beneficial for this agent to act on a highly conserved site to increase its therapeutic lifespan. Recently, work by us and others have described novel human monoclonal antibodies capable of very broad heterotypic protection that could be used in the treatment and prevention of influenza virus infections [12], [13], [14]. Here we report *in vitro* neutralization and *in vivo* efficacy in prophylactic and therapeutic mouse models of the novel 2009 H1N1 pandemic influenza virus infection by one such broadly protective antibody derived from an H5N1 avian influenza survivor.

## Methods

### Antibody expression and purification

Human IgG1 antibody was expressed and purified essentially as previously described [12].

### Preparation of virus stocks

The A/California/04/2009 virus used in the microneutralization studies is a recombinogenic virus composed of the hemagglutinin (HA) and neuraminidase (NA) gene segments from A/California/04/2009 and the remaining influenza viral gene segments are from A/PR/8/34 [15]. The recombinant virus was propagated in MDCK cell culture. All other strains were amplified in 10–11 day old embryonated hens' eggs.

### Microneutralization assay

Microneutralization assays were performed as previously described [12]. Briefly, two-fold dilutions of mAb were incubated with 100 TCID_50_ of virus for 1 h at 37°C prior to addition to monolayers of MDCK cells. Cell monolayers were incubated for 72 h, and the presence of virus in supernatant was determined by HA assay of duplicate samples. The neutralizing titer was defined as the minimum inhibitory concentration at which the infectivity of 100 TCID_50_ of the appropriate virus for MDCK cells was completely neutralized in duplicate wells.

### mAbs for prophylaxis and therapy in mice

Animal experiments were performed in accordance with the guidelines of the Mount Sinai School of Medicine and St. Jude Children's Research Hospital Institutional Animal Care and Use Committees (IACUC).

Female 6–8 weeks old Balb/C (Jackson Laboratories) or DBA/2 (Charles River) mice were housed 5–6 per cage in ABSL3+ containment. Food and water were provided *ad libitum*. For the prophylactic studies, mice (5–6 per group, except where noted) received 1, 2.5, 10, or 25 mg antibody A06 per kg of bodyweight in approximately 200–300 µL of sterile phosphate-buffered saline (PBS) by intraperitoneal (IP) injection. The control groups received 200–300 µL of 25 mg/kg non-immune human serum IgG (Sigma) (n = 3) or PBS by IP injection. Antibody was administered either 1 hour (Balb/C) or 24 hours (DBA/2) before being challenged with A/California/04/09, which had been previously mouse-adapted by 9 sequential lung passages, or wild-type A/Netherlands/602 virus. Mice were inoculated by intranasal administration of 3.3, 25, or 33 MLD_50_ (50% mouse lethal dose) influenza virus in 30–50 µL of PBS. 2000 PFU (25 MLD_50_) of mouse-adapted A/California/04/09 was used for infection of Balb/C mice in the prophylactic and therapeutic studies, while 10 PFU (3.3 MLD_50_) and 100 PFU (33 MLD_50_) was used for the A/Netherlands/602 strain in DBA.2 mice. Symptoms preceding death are weight loss >30% and general inactivity. Morbidity and mortality were monitored either daily or at days 0, 3, 7, 10, and 14.

For therapeutic studies, Balb/C mice (10 per group) were given a lethal virus dose of 25 MLD_50_ A/California/04/09 (2000 PFU) followed by a single 15 mg/kg dose of antibody 24, 48, 72, 96, 120, or 144 hours post infection. Morbidity and mortality were monitored for 17 days and the mice were weighed on days 0, 3, 7, 10, 14, and 17 following virus challenge. For dose escalation studies, mice were given a viral dose of 3.3 MLD_50_ (10 PFU) A/Netherlands/602/209 followed either 1 day or 2 days post infection with a single IP injection of 2.5, 10 or 25 mg/kg dose of antibody A06, vehicle (PBS), or non-immune IgG (25 mg/kg dose). Morbidity and mortality were monitored for 14 days and the mice were weighed daily following virus challenge.

All data for both the prophylactic and therapeutic studies was plotted for days 3, 7, 10, 14, and 17 (where appropriate). Survival data were plotted (Kaplan-Meier) and analyzed using the logrank test to determine statistical significance (P<0.05). Mean weight data were also plotted. All data were plotted and analyzed using GraphPad Prism v.5.02 software.

### 2009 novel H1N1 predicted antibody binding site sequence analysis

One thousand non-redundant 2009 novel H1N1 hemagglutinin amino acid sequences deposited to the Influenza Sequence Database [16] were aligned using MUSCLE v4 multiple sequence alignment function accessible through the Influenza Sequence Database website (http://www.ncbi.nlm.nih.gov/genomes/FLU/FLU.html). Sequences were visually inspected for amino acid changes within the predicted antibody binding site in the hemagglutinin HA2 region described in [13], [17]. Variants in both the predicted contacting and non-contacting positions were noted along with the frequency of occurrence.

## Results

### Pandemic H1N1 virus neutralization *in vitro* by antibody A06

We previously reported the discovery of broadly neutralizing antibodies from avian influenza survivor antibody libraries, capable of mechanistically novel, heterosubtypic neutralization against numerous H1N1 and H5N1 viruses [12]. Subsequent to our publication, others have reported highly related and broadly neutralizing human antibodies [13], [14]. The novel unifying mechanism these anti-hemagglutinin neutralizing antibodies exhibit is that they do not inhibit virus-induced hemagglutination. Structural analysis by both Sui et al. and Ekiert et al. have shown the antibodies bind to the highly conserved stem of hemagglutinin (HA) that prevents a conformational change required for viral host cell fusion [13], [17]. The reason these antibodies are broadly neutralizing is attributed to the high sequence conservation of the antibody epitope between H1, H5 and H9 type hemagglutinins, which is coincidentally maintained in the newly emergent 2009 pandemic H1N1 strain (Table 1). From these collective observations, we predicted the 2009 pandemic H1N1 influenza would be susceptible to neutralization by the previously described antibody isolated from the Turkish avian influenza survivor libraries.

**Table 1:** The A-helix epitope targeted by the A06 antibody is highly conserved across numerous types of influenza.

Subtype	Strain	18	19	20	21	40	41	42	43	44	45	46	47	48	49	50	51	52	53	54	55	56	MIC
Novel H1N1	A/California/04/2009 6:2	V	D	G	W	S	T	Q	N	A	I	D	E	I	T	N	K	V	N	S	V	I	<10
H1N1	A/South Carolina 1918	I	-	-	-	-	-	-	-	-	-	-	G	-	-	-	-	-	-	-	-	-	ND
	A/Puerto Rico/8/34	I	-	-	-	-	-	-	-	-	-	N	G	-	-	-	-	-	-	T	-	-	62-125
	A/New Caledonia/20/99	-	-	-	-	-	-	-	-	-	-	N	G	-	-	-	-	-	-	-	-	-	9*
	A/Solomon Islands/3/2006	-	-	-	-	-	-	-	-	-	-	N	G	-	-	-	-	-	-	-	-	-	83
	A/Brisbane/59/2007	-	-	-	-	-	-	-	-	-	-	N	G	-	-	-	-	-	-	-	-	-	24
	A/Texas/36/1991	I	-	-	-	-	-	-	-	-	-	N	G	-	-	-	-	-	-	-	-	-	250
H5N1	A/Indonesia/5/05	-	-	-	-	-	-	-	K	-	-	-	G	V	-	-	-	-	-	-	I	-	9*
	A/Vietnam/1203/04	-	-	-	-	-	-	-	K	-	-	-	G	V	-	-	-	-	-	-	I	-	11*
	A/Egypt/14725/06	-	-	-	-	-	-	-	K	-	-	-	G	V	-	-	-	-	-	-	I	-	2*
	A/Turkey/65596/2006	-	-	-	-	-	-	-	K	-	-	-	G	V	-	-	-	-	-	-	I	-	9*
H9N2	A/Hong Kong/1073/99	-	A	-	-	-	-	-	K	-	-	-	K	-	-	S	-	-	-	N	I	V	ND

A-helix epitope sequences from novel H1N1, current and past seasonal isolates of H1N1, H5N1 and avian H9N2 hemagglutinin proteins were aligned. Positions are labeled according to HA2 numbering. Amino acids at positions 19-21, 41, 42, 45, 46, 49, 52, 53 and 56 are antibody contact points [13], [17]. The column on the far right indicates *in vitro* microneutralization minimal inhibitory concentrations (MIC) of antibody A06 in µg/ml for the isolates tested (ND = not done, * = previously reported data [8]).

As a first step to test our prediction the A06 antibody (previously referred to as mAb1 [12]) was tested in *in vitro* viral microneutralization assays against a recombinogenic virus containing the 2009 H1N1 pandemic reference isolate A/California/04/2009 influenza virus (CA04) HA and neuraminidase (NA) proteins upon a A/PR/8/34 based viral background, (hereafter referred to as A/California/04/2009 6:2) [18]. In these assays the A06 antibody demonstrated complete viral neutralization of the 2009 H1N1 virus at final concentrations as low as 10 µg/ml (Table 1), which is in good agreement with neutralization results against other H5N1 and H1N1 strains that we have tested (Table 1).

### Antibody A06 *in vivo* prophylaxis against the 2009 pandemic virus

**Figure 1:**
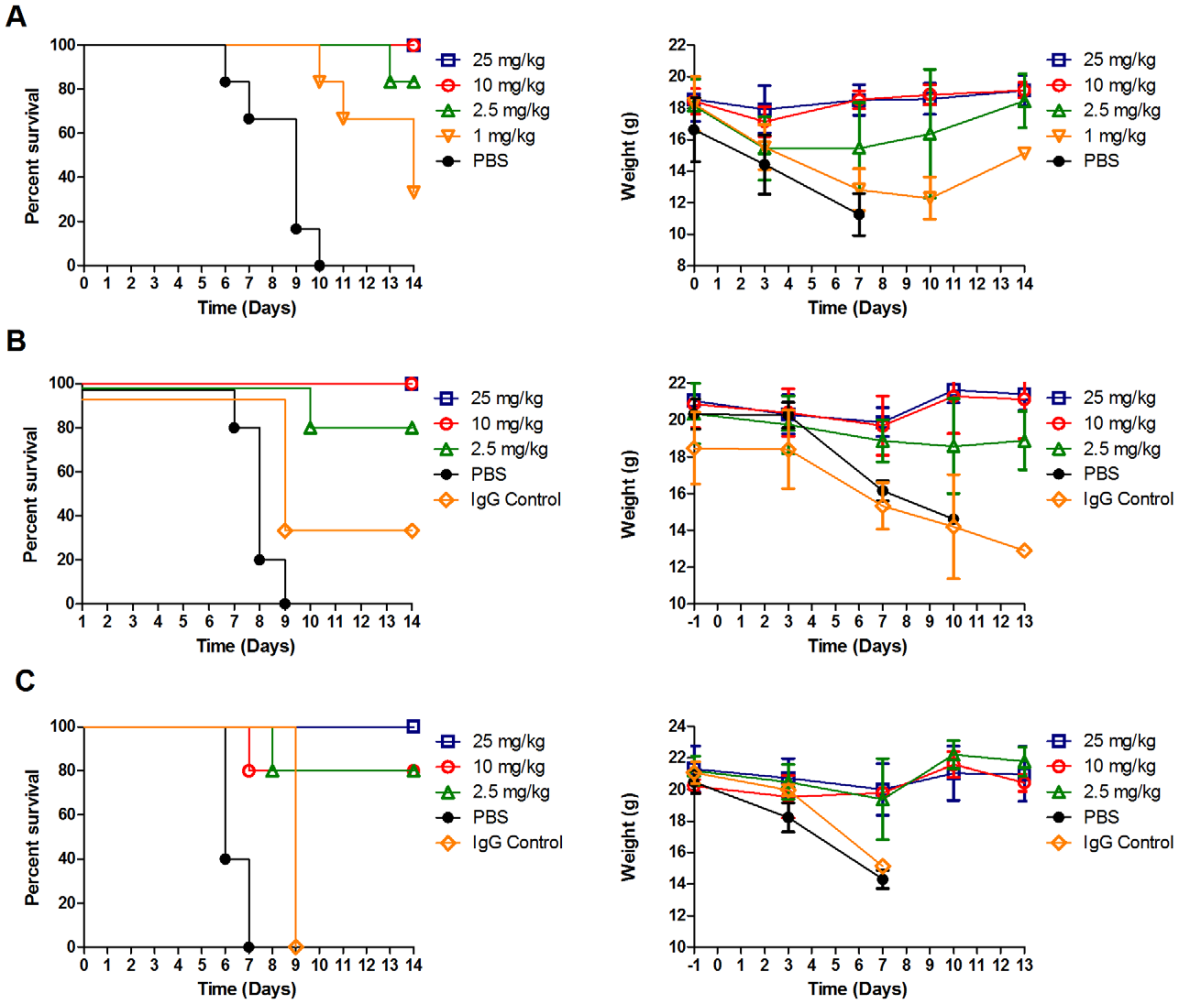
Antibody A06 prophylaxis protects mice from a lethal 2009 pandemic H1N1 influenza virus challenge. (A) Balb/C mice (n = 6 except where noted) were challenged with 25MLD50 of a mouse-adapted 2009 H1N1 pandemic influenza A/California/04/2009 reference isolate 1 hour after a single interperitoneal injection of the indicated dose of A06 antibody. Survival (left panel) and weight (right panel) were monitored over a 14 day period. Open blue squares- 25 mg/kg antibody A06, open red circles- 10 mg/kg antibody A06 (n = 5), open green triangles- 2.5 mg/kg antibody A06, open orange triangles- 1 mg/kg antibody A06, and black filled circles- PBS vehicle control. (B) and (C) DBA/2 mice (n = 5 except where noted) were challenged with either 3.3MLD50 (B) or 33MLD50 (C) A/Netherlands/602/2009 H1N1 pandemic influenza reference isolate 24 hours after a single interperitoneal injection of the indicated dose of antibody A06. Survival (left panel) and weight (right panel) were monitored over 13 days post-infection. Open blue squares- 25 mg/kg antibody A06, open red circles- 10 mg/kg antibody A06, open green triangles- 2.5 mg/kg antibody A06, black filled circles- PBS vehicle control, open orange diamonds- 25 mg/kg human IgG control (n = 3).

To assess whether the A06 antibody could prevent or decrease the severity of influenza infection *in vivo*, we performed a dose-escalating study in a murine prophylactic model of disease. Briefly, Balb/C mice were given a single IP dose of A06, infected intranasally 1 hour later with 25MLD_50_ of a mouse-adapted CA04 H1N1 virus (see methods), and then monitored for survival and body weight over the following 14 days (Figure 1A). In this study, vehicle treated mice either died or were euthanized 6 to 10 days post-infection and displayed pronounced progressive weight loss during the course of infection. In sharp contrast, mice treated with either 25 mg/kg or 10 mg/kg of A06 survived the lethal challenge and regained lost weight by day 7. Survival in the 2.5 mg/kg treatment group was 83%, with the mice losing more weight than the higher dosed groups in the first 3 days post-infection, but still regaining their pre-study weights by day 14. Survival in the 1 mg/kg group was observed, but was the least prominent of all treatment groups (33%) with the surviving mice losing body weight through 10 days post-infection. Logrank test analysis of the survival curves demonstrated statistical significance (P<0.0001). These studies demonstrate antibody A06 is able to protect mice from the lethality and weight loss associated with influenza virus infection in a dose dependent manner.

To further support the previous results, we wanted to show efficacy on a non-mouse- adapted novel human H1N1 strain as well as assess efficacy against two different levels of viral challenge. In the subsequent prophylaxis study, we used a novel H1N1 influenza strain A/Netherlands/602/2009 (Netherlands602) in the more sensitive and susceptible DBA/2 mouse strain at both 3.3MLD_50_ (Figure 1B) and 33MLD_50_ (Figure 1C). In the 3.3MLD_50_ challenged study, mice treated with vehicle died or were euthanized between 7 and 9 days post infection and displayed pronounced progressive weight loss during the course of infection. Antibody A06 administration provided significant survival (P<0.0001) and considerable body weight maintenance benefits. Specifically, mice challenged with 3.3MLD_50_ Netherlands602 in both the 25 mg/kg and 10 mg/kg dose groups survived and lost some weight through day 7 that was rapidly regained to their pre-study levels by day 10. Survival in the 2.5 mg/kg treatment group was 80% with greater weight loss observed compared to the higher dosed groups.

In the subsequent DBA/2 study where mice were challenged with 10 times more virus (33MLD_50_) than the previous A06 antibody treated groups they also displayed significant survival (P<0.0001)and substantial body weight maintenance benefits compared to controls. Specifically, the mice manifested disease and mortality more rapidly than those in the 3.3MLD_50_ challenge study, as both groups treated with PBS or non-immune IgG died or were euthanized between 6 and 7 days post infection. In contrast, all mice treated with 25 mg/kg of antibody A06 survived, whereas those treated with 10 mg/kg or 2.5 mg/kg of A06 had an 80% survival rate. The average body weight of all the treated groups declined through the first 7 days post-infection, but was restored to pre-study levels by day 10. In summary, prophylactic administration of antibody A06 appeared beneficial in abrogating influenza-mediated weight loss, allowing faster recovery of infected animals.

### Therapeutic activity of antibody A06 in 2009 pandemic H1N1 infection

**Figure 2:**
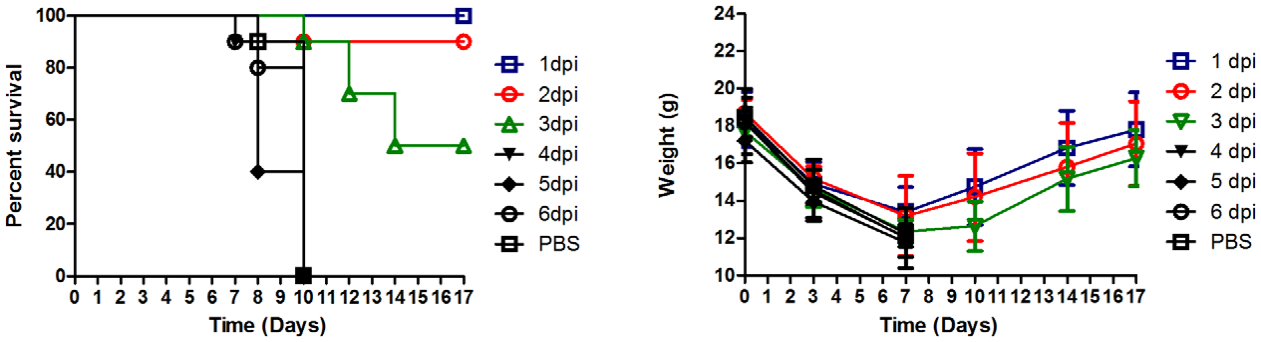
Antibody A06 therapy protects Balb/C mice from death by 2009 pandemic H1N1 influenza infection. Balb/C (n = 10, except groups 4dpi and PBS where n = 9) were infected with 25MLD50 A/California/04/2009. A single administration of 15 mg/kg per group was given 1–6 days post-infection. Open blue squares- 1 day post infection, open red circles- 2 days post- infection, open green triangles- 3 days post-infection, filled triangles- 4 days post- infection, filled diamonds- 5 days post-infection, open black circles- 6 days post- infection, and open black squares- PBS vehicle 1 day post-infection. Survival (left panel) and weight (right panel) were monitored for 17 days after infection.

Passive immunity may provide both prophylactic and therapeutic benefits against influenza infection. To address whether the A06 antibody is therapeutically effective following infection, we treated groups of CA04-infected Balb/C mice (25MLD_50_ infectious titer) with a single 15 mg/kg dose of antibody A06 at 1, 2, 3, 4, 5, or 6 days post-infection. All mice dosed 1 day after infection survived, 90% of mice dosed 2 days after infection survived, and 50% of the mice dosed 3 days after infection survived. All mice dosed 4 days post infection and later either died or were euthanized between days 7 and 10. Weight loss in the therapeutic study was more severe than seen in the mice treated prophylactically and similar to the vehicle-treated mice in the prophylaxis study. All mice in this study lost ∼30% of their body weight due to the established novel H1N1 infection (Figure 2B) and earlier treatment appeared linked to higher study end weights and survival (Figure 2A and B). These results demonstrate the utility of a therapeutic passive immunity approach against an emergent strain of influenza and extend previous findings that heterotypic neutralizing antibodies are beneficial in *in vivo* models of influenza prophylaxis and therapy.

**Figure 3:**
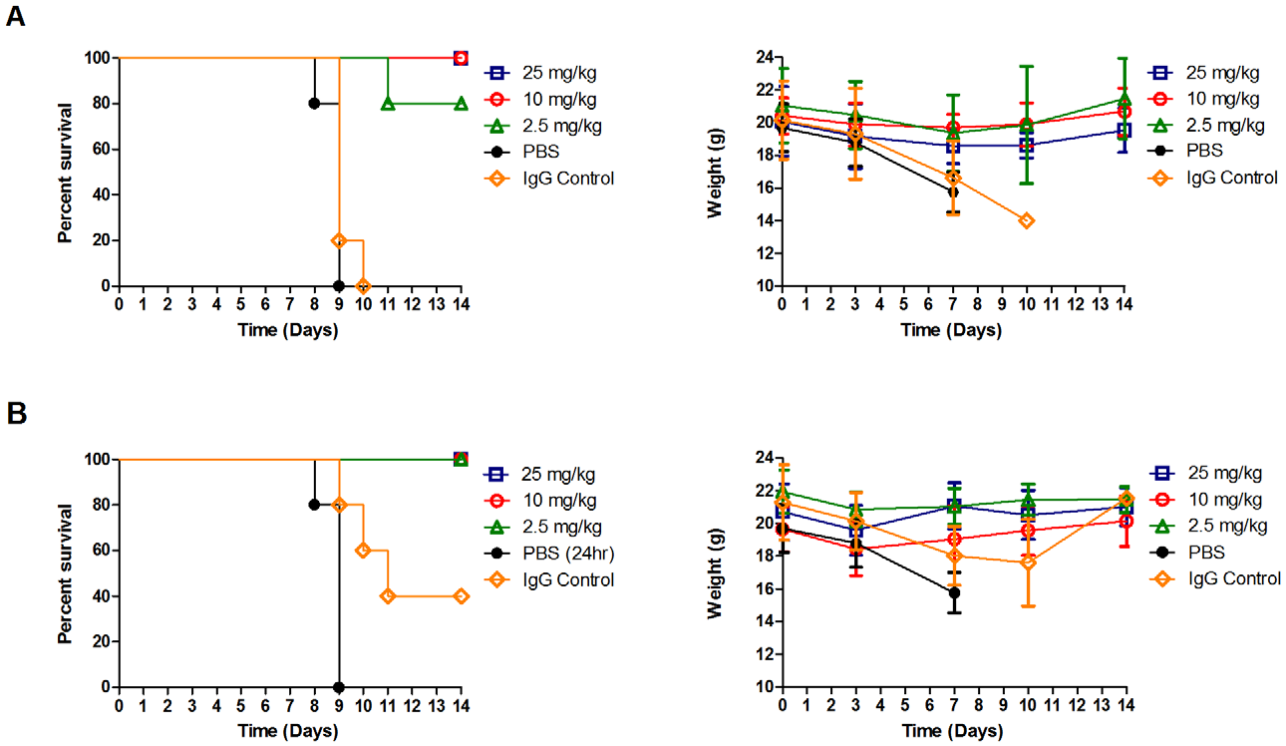
Antibody A06 therapy protects DBA/2 mice from death by 2009 pandemic H1N1 influenza infection. DBA/2 mice were infected with 3.3MLD50 of A/Netherlands/602/2009 and treated with a single administration of antibody A06 1 day (A) or 2 days (B) post-infection. Three different A06 concentrations were tested along with vehicle (PBS) and non-specific IgG controls. Animals were monitored for survival (left panels) and weight (right panels) over a 14 day period. Treatment groups (n = 5) are labeled as in Figure1B and 2C. Open blue squares- 25 mg/kg antibody A06, open red circles- 10 mg/kg antibody A06, open green triangles- 2.5 mg/kg antibody A06, black filled circles- PBS vehicle control, open orange diamonds- 25 mg/kg human IgG control.

As a significant benefit in overcoming influenza infection was seen in treatment groups administered A06 antibody at 1 or 2 days post infection, our next study expanded the analysis at these times through a dose escalation study. Specifically, we administered the A06 antibody at 2.5, 10, and 25 mg/kg either 1 day (Figure 3A) or 2 days (Figure 3B) after infection with 3.3MLD_50_ of the Netherlands602 strain of the 2009 pandemic H1N1 virus in DBA/2 mice. As seen previously, PBS vehicle treated mice died or were euthanized by 9 days post infection and displayed pronounced progressive weight loss during the course of infection. However, mice receiving 25 mg/kg or 10 mg/kg doses of antibody A06, either at 1 day and 2 days post infection, survived the Netherland602 virus infection, corroborating results found with the CA04 viral challenge (Figure 3A and 3B, left panels). Importantly, the lowest antibody dose (2.5 mg/kg) was sufficient to overcome infection in all except one animal. As a benefit the treated mice also gained weight after treatment with A06, arriving at their pre-study weight by day 14 (Figure 3A and 3B, right panels). In summary, our results demonstrate that antibody A06 is a very effective treatment following novel H1N1 infection, even at doses of 2.5 mg/kg in two different mouse models of influenza infection and treatment.

### Conservation of the predicted antibody epitope in novel H1N1 isolates

Broadly active anti-influenza agents need to target essential sites that are minimally prone to mutation. As a predictive assessment of the potential efficacy of the A06 antibody against current H1N1 pandemic isolates, we analyzed a large number of novel influenza hemagglutinin protein sequences within the proposed A-helix epitope [13], [17] for genetic drift. From the analysis of 1000 full length hemagglutinin protein sequences (NCBI Influenza Virus Resource November 11, 2009) [16], we found only 5 isolates that varied from CA04 reference strain at three positions in the proposed A-helix antibody epitope on the HA2 subunit (Table 2). Only one of the five isolates (Canada-NS/RV1535/2009) contained a mutation to a proposed contact point, which was a conservative substitution of valine for isoleucine at residue 56. It is significant to point out that isoleucine is found at an analogous position in the H9N2 Hong Kong/1073/99 which is recognized by the A06 antibody (Table 1 and unpublished data), suggesting the isolate would still be susceptible to the A06 antibody. Three of the remaining four mutations occurred at the non-contacting residue 43, where lysine, serine, or aspartic acid was found instead of asparagine. The final mutation was a proline replacement for alanine non-contacting position 44. In summary, the analysis suggests the isolates display limited allowances for genetic drift within this region that may maintain susceptibility to A06.

**Table 2:** Sequence analysis of novel H1N1 HA isolates shows limited variation in the predicted neutralization epitope.

Accession	Strain	18	19	20	21	40	41	42	43	44	45	46	47	48	49	50	51	52	53	54	55	56	Frequency
ACS45035	A/California/04/2009	V	D	G	W	S	T	Q	N	A	I	D	E	I	T	N	K	V	N	S	V	I	98.70%
ACS34967	A/Sakai/2/2009	-	-	-	-	-	-	-	K	-	-	-	-	-	-	-	-	-	-	-	-	-	0.30%
ACY46863	A/Singapore/GP2687/2009	-	-	-	-	-	-	-	S	-	-	-	-	-	-	-	-	-	-	-	-	-	0.20%
ACY26192	A/Malaysia/820/2009	-	-	-	-	-	-	-	D	-	-	-	-	-	-	-	-	-	-	-	-	-	0.10%
ACV67229	A/Utah/06/2009	-	-	-	-	-	-	-	-	P	-	-	-	-	-	-	-	-	-	-	-	-	0.10%
ACQ73385	A/Canada-NS/RV1535/2009	-	-	-	-	-	-	-	-	-	-	-	-	-	-	-	-	-	-	-	-	V	0.60%

Alignment of hemagglutinin protein from novel H1N1 isolates deposited in the NCBI Influenza Virus Resource was performed using the multiple sequence alignment application within the database. 1000 full length HA sequences contained in the database on November 11, 2009 were analyzed for variation in HA2 residues previously shown to be contacted by neutralizing antibodies binding to the stem region [13], [17].

## Discussion

We previously demonstrated, *in vitro*, that the A06 antibody neutralizes a broad range of seasonal H1N1 and avian H5N1 influenza viruses causing human disease. In this study, we extend these results and demonstrate that the A06 antibody is able to protect from and treat the antigenically distinct 2009 pandemic H1N1 virus infection in mouse models and also neutralize the current seasonal H1N1 Brisbane/59/2007 strain *in vitro*. These results provide further evidence for the use of passive immunity as a weapon against influenza infection.

Passive immunity offers several benefits in comparison to current chemotherapeutic anti-viral treatment options. First, passive immunity provides the opportunity to protect at-risk individuals from infection. At-risk segments of the population include those who do not mount an immune response to vaccine, the immunocompromised, those in poor health, pregnant women, and those in critical care. The potential for long-lasting protection arising from a single injection of antibodies such as A06 is appealing. In addition, while orally available drugs are desirable to reach a larger patient population and increase patient compliance in courses of treatment, their use in critical care settings involving the later stages of disease is limited by the route of administration. Quite simply, injectable therapies are needed for patients that are unable to receive orally administered anti-influenza treatment.

Current anti-viral treatments provide ease of use and therapeutic benefit early in the course of infection. However, they suffer from several limitations, namely high rates of resistance, as exhibited recently in the seasonal H1N1 virus [19]. The unexpected speed at which the H274Y mutation conferring oseltamivir resistance took over as the dominant strain in the 2007–2008 influenza season demonstrates the challenges facing widespread use of anti-viral agents targeting the neuraminidase protein [3], [19], [20]. Antibodies such as A06 that attack a highly conserved region of the hemagglutinin protein and not the mutagenic hot spots near the receptor binding domain or the neuraminidase protein may face fewer challenges arising from mutation. Using the method of Caton, et al [21], we have not been able to generate escape mutants after multiple attempts using the A06 antibody on both H1N1 and H5N1 influenza strains, suggesting that A06 is attacking a conserved, susceptible portion of the virus (JS, unpublished results). Furthermore, conservation of the predicted antibody binding site in the 2009 pandemic strain isolates demonstrates the epitope has not changed significantly from the time of its emergence in March 2009. It is likely the ability to tolerate change in the hemagglutinin A-helix/fusion peptide region may be highly restricted due to functional constraints, as evidenced by the maintenance of this epitope across numerous influenza sub-types.

However, an alternative interpretation to this observation is that the region has not been sufficiently pressured to change and may mutate when subjected to greater selective pressure, even though we have not seen it yet in escape mutant analysis. Nevertheless, even if escape were possible, passive immunization would likely be highly effective when used in conjunction with other established therapies to reduce the prospect of viral escape or resistance.

Here we have presented A06 antibody in vitro neutralization results with numerous H1N1 and H5N1 strains from each sub-type. Though recent H1N1 strains were neutralized with similar antibody concentrations, two older strains, A/PR/8/34 and A/Texas/1991, required substantially higher amounts of antibody to be effective. Upon further sequence examination of these two recent strains we have observed potential N-linked glycosylation sites proximal to the predicted epitope in A/PR/8/34 (amino acids 285–287) and A/Texas/1991 (amino acids 286–288). It is possible that glycosylation at theses sites sterically hinders the antibody and reduces its efficacy in this in vitro system. Further testing will need to be performed both *in vitro* and *in vivo* to evaluate the relevance of such a potential glycosylation site. Still, all H5N1 strains tested, representing all major clades of highly pathogenic avian influenza, were effectively neutralized by antibody A06. Considering the ability of the antibody to neutralize the novel H1N1 virus, multiple seasonal H1N1 isolates, isolates from all clades of human H5N1, and that the proposed epitope is highly conserved amongst the initial sampling of one thousand reported novel H1N1 hemagglutinin isolates, we predict that A06 will be active against influenza strains bearing this epitope.

Additional testing is required to determine the efficacy and utility of passive immunity in man. However, the profile of the A06 antibody and other broadly protective antibodies warrants their testing in man. Success of such antibodies would justify their use in cases of local, national, and global crisis. In addition, injectable administration of antibodies such as A06 could protect critical care patients unable to receive orally- administered anti-viral therapy. Use of passive immune therapy in an integrative approach with anti-viral chemotherapeutics could even decrease the frequency and speed at which resistance to either agent is generated. Furthermore, as these types of antibodies were found in large survivor, vaccinee, and naïve donor combinatorial antibody libraries, it suggests the mode of activity is immunologically relevant. As a result, these broadly reactive anti-fusion antibodies and their protective mechanisms should also be used as an additional guide in the production and assessment of all future influenza vaccines.
